# Production of Cloned Miniature Pigs Expressing High Levels of Human Apolipoprotein(a) in Plasma

**DOI:** 10.1371/journal.pone.0132155

**Published:** 2015-07-06

**Authors:** Masayuki Ozawa, Takehiro Himaki, Shoji Ookutsu, Yamato Mizobe, Junki Ogawa, Kazuchika Miyoshi, Akira Yabuki, Jianglin Fan, Mitsutoshi Yoshida

**Affiliations:** 1 Department of Biochemistry and Molecular Biology, Graduate School of Medical and Dental Sciences, Kagoshima University, Kagoshima, Japan; 2 Laboratory of Animal Reproduction, Faculty of Agriculture, Kagoshima University, Kagoshima, Japan; 3 Laboratory of Veterinary Clinical Pathology, Joint Faculty of Veterinary Medicine, Kagoshima University, Kagoshima, Japan; 4 Department of Molecular Pathology, Interdisciplinary Graduate School of Medicine and Engineering, University of Yamanashi, Yamanashi, Japan; Katholieke Universiteit Leuven, BELGIUM

## Abstract

High lipoprotein(a) [Lp(a)] levels are a major risk factor for the development of atherosclerosis. However, because apolipoprotein(a) [apo(a)], the unique component of Lp(a), is found only in primates and humans, the study of human Lp(a) has been hampered due to the lack of appropriate animal models. Using somatic cell nuclear transfer (SCNT) techniques, we produced transgenic miniature pigs expressing human apo(a) in the plasma. First, we placed the hemagglutinin (HA)-tagged cDNA of human apo(a) under the control of the β-actin promoter and cytomegalovirus enhancer, and then introduced this construct into kidney epithelial cells. Immunostaining of cells with anti-HA antibody allowed identification of cells stably expressing apo(a); one of the positive clones was used to provide donor cells for SCNT, yielding blastocysts that expressed apo(a). Immunohistochemical analysis of tissue sections and RT-PCR analysis of total RNA from organs of cloned piglet revealed that apo(a) is expressed in various tissues/organs including heart, liver, kidney, and intestine. More importantly, a transgenic line exhibited a high level (>400 mg/dL) of Lp(a) in plasma, and the transgenic apo(a) gene was transmitted to the offspring. Thus, we generated a human apo(a)–transgenic miniature pig that can be used as a model system to study advanced atherosclerosis related to human disease. The anatomical and physiological similarities between the swine and human cardiovascular systems will make this pig model a valuable source of information on the role of apo(a) in the formation of atherosclerosis, as well as the mechanisms underlying vascular health and disease.

## Introduction

High plasma levels of Lp(a) are associated with human cardiovascular disease, including coronary heart disease, stroke, and restenosis [1–3]. The Lp(a) particle closely resembles low-density lipoprotein (LDL) in its lipid composition. Moreover, like LDL, Lp(a) also contains apolipoprotein B-100 (apoB-100), but is distinguished by the presence of an additional glycoprotein known as apolipoprotein(a) [apo(a)]. Apo(a) has a remarkable homology to plasminogen [4], but its function remains elusive. Although progress has been made in elucidating the Lp(a) phenotype and genotype, the *in vivo* functions of Lp(a) have not yet been fully elucidated.

The major difficulty in defining the *in vivo* functional roles of Lp(a) in atherosclerosis is the lack of appropriate experimental animals [5]. This scarcity of animal models is a result of the fact that Lp(a) occurs naturally only in Old World monkeys and humans. The development of transgenic animals expressing human apo(a) has provided an alternative means to study of Lp(a) functions. To date, transgenic human apo(a) animal models have been produced in mice and rabbits [6–9]. However, it is difficult to translate findings from small rodent animal models to humans because of their major differences in physiology. Furthermore, the plasma lipoprotein profile of rabbits is somewhat different from that of humans [10].

By contrast, pigs have a cardiovascular system that is similar to that of humans, and therefore have the potential to serve as an effective animal model for cardiovascular disease [11]. The pig model is more effective for studying the relationship between lipoprotein metabolism and atherosclerosis, because pigs have both LDL and high-density lipoprotein (HDL) particles circulating in plasma [12,13]. Furthermore, pigs and humans are highly similar with respect to lipid metabolism and cardiovascular physiology [14,15], and pig models of induced coronary artery disease exhibit a natural disease progression similar to that observed in human patients [16]. In particular, the Clawn miniature pigs used in this study are inbred and weigh a maximum of 60–80 kg [17], and their organs are the same size as those of humans. In addition, the haplotypes of the swine leukocyte antigens, which are important immunogens for humoral responses and important mediators of cellular immune responses, are well defined in these miniature pigs [18]. Together, these features make transgenic Clawn miniature pigs useful animals for the development of human disease models.

In the present study, we produced a cloned Clawn miniature pig expressing high levels of human apo(a) in plasma through the transfection of somatic cells combined with nuclear transfer. The studies using the cloned pig will contribute to understanding of the mechanism(s) by which higher levels of Lp(a) affect the development of human cardiovascular disease.

## Materials and Methods

### Ethics statement

Experiments with recombinant DNA technology were performed in agreement with the guidelines of Kagoshima University Committee on recombinant DNA security. All animal experiments in this study were approved by the Animal Ethics Committee, Kagoshima University. The committee defines the timing to give humane endpoints and the method of sacrifice. The criteria used in the present study to determine when the animals should be humanely sacrificed include following activities. 1) Whether it can move? 2) Whether it can eat (drink milk)? 3) Body temperature. Immediately after cesarean section, we carried out the veterinary care for maintenance of body temperature, moisture retention, nutrient supply and oxygen supply of miniature clone piglets using the neonatal incubator. In the first series of experiments, when piglets stopped drinking milk and moving, and their body temperature become low, we decided to sacrifice them. Throughout the experiments using animals, there was veterinary care. In the first series of experiments, after birth pigs were monitored directly every 30 min. In the second series experiments, pigs were monitored every one-hour using an Internet camera equipped in the house and monitored directly by the technicians of the house. Before administration of pentobarbital to sacrifice the piglets, neither analgesics nor anaestetics was used.

### Vector construction

**Fig 1:**
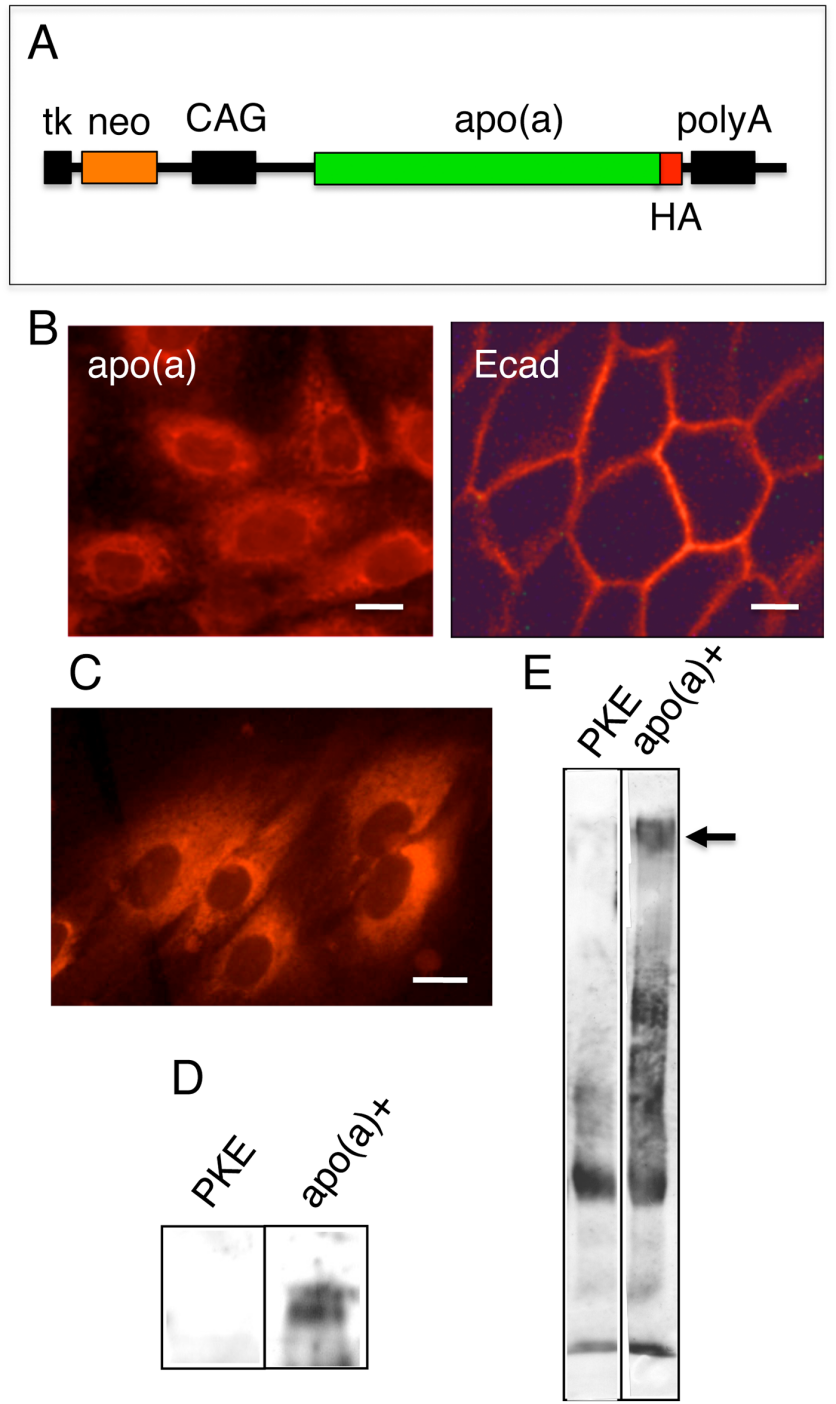
Isolation of pig kidney epithelial cells stably expressing human apo(a). A, schematic representation of the pC-apo(a)HA construct. The vector contains the CAG promoter, HA-tagged apo(a) gene, β-globin polyadenylation signal (polyA), and neo gene under the control of herpes simplex virus thymidine kinase (tk) promoter. B, stable transfectants of MDCK cells expressing HA-tagged human apo(a) or HA-tagged E-cadherin (Ecad) were immunostained with anti-HA monoclonal antibody, followed by rhodamine-conjugated anti-rat IgG. Note the punctate staining of HA fluorescent signals that correspond to ER in apo(a)-expressing cells and the membrane staining of the cell–cell contact sites in E-cadherin (a cell–cell adhesion molecule)-expressing cells. Bar, 25 μm. C, Stably transfected pig kidney epithelial cells expressing human apo(a) were immunostained with anti-HA monoclonal antibody. Note the ER staining. Bar, 25 μm. D, Total cell lysates of parental pig kidney epithelial (PKE) cells or stable transfectants [apo(a)+] were subjected to immunoblot analysis with anti-HA antibody. E, Conditioned medium of PKE or apo(a)+ cells were subjected to immunoprecipitation with anti-HA antibody. Collected materials were subjected to immunoblot analysis with anti-apo(a) antibody.

The plasmid vector used in this experiment, pC-apo(a)HA, is shown in Fig 1. Human apo(a) cDNA has been described [4]. To facilitate the detection of apo(a), apo(a) was tagged with hemagglutinin (HA) at the *C*-terminus by PCR using the primers ATCCCTCTCTGTGCATCCTCT and CGAATTATTTCTCATCATTCCCTCAA and apo(a) cDNA as a template. After generating blunt ends using T4 DNA polymerase, the PCR product was digested with the *Pvu*I restriction enzyme and cloned into the *Eco*RV site of the pC-SnailHA vector [19]. Then, the *Eco*RI–*Kpn*I fragment of apo(a) was cloned into the vector, yielding pC-apo(a)HA.

### Preparation of pig kidney epithelial cells and transfection

Primary kidney epithelial cells were isolated from the kidney tissue of a Clawn miniature boar as described in [20]. Growing cell colonies were subcultured for several generations, and then frozen until needed. Prior to use in experiments, frozen cells were thawed and passaged for 3–7 generations before transfection. Transfection of kidney epithelial cells with the pC-apo(a)HA plasmid was performed using the Amaxa Nucleofector system (Amaxa GmbH, Cologne, Germany) as described previously [21]. After transfection, cells were incubated in culture medium at 37°C. Five days later, cells were incubated with medium containing 200 μg/ml of G418 (Geneticin; Invitrogen, Carlsbad, CA, USA) for an additional 10–15 days to isolate drug-resistant colonies. Colonies (comprising 300–700 cells) were picked using Pipetman tips and directly transferred to individual wells in 24-well plates containing 1 ml culture medium with G418. The cells were cultured for 6–10 more days, and then a portion of each colony was analyzed for apo(a) expression by immunofluorescence staining with anti-HA antibodies. Homogeneous apo(a) positive clones were selected and expanded; 1 week later, they were assayed to confirm stability and homogeneity of apo(a) expression, and then stored frozen.

### Antibodies

A rat mAb against HA was purchased from Roche Applied Science (Mannheim, Germany). A goat anti-apo(a) (anti-lipoprotein(a)) antibody was purchased from Applied Biological Materials (Richmond, BC, Canada). A mouse anti-apo(a) mAb (anti-LPA, 4H1) was obtained from GeneTex, Inc (Irvine, CA, USA). Mouse anti–E-cadherin antibody and anti-cytokeratin 5 and 8 were obtained from Transduction Laboratories (Lexington, KY, USA) and Chemicon International (Temecula, CA, USA), respectively. A chicken anti-apoB mAb, which recognizes apoB of multiple species, was obtained from Hiroshima Bio-medical Co. Ltd. (Hiroshima, Japan). All secondary antibodies were obtained from Jackson ImmunoResearch Laboratories (West Grove, PA, USA).

### Production and activation of SCNT embryos

SCNT miniature pig embryos were produced as described previously [22] and activated by ultrasound stimulation [23]. Briefly, oocytes with a polar body were treated with 0.5 μg/ml demecolcine (Sigma-Aldrich Japan (Tokyo, Japan) and 20 mM sucrose for 0.5–1 h. Enucleation was performed by aspirating the first polar body and protruding membrane using a 15 μm inner diameter glass pipette. After enucleation, a single donor cell was inserted into the perivitelline space of each oocyte using the same glass pipette. Membrane fusion of the cytoplast and donor cell was induced by applying a single direct-current pulse [22]. Following the fusion pulse, the complexes were cultured for 2 h under 5% CO_2_, 5% O_2_, and 90% N_2_ at 38.5°C, in a 100 μl droplet of modified PZM-3 (mPZM-3) [22] until activation. Fusion status was determined by microscopic examination 1.5 h after applying the pulse. In the first series of experiments, the embryos were cultured in 50 μl of modified porcine zygote medium-3 (mPZM-3) supplemented with 2.2 μg/ml cytochalasin B [22], and then incubated for 2 h under 5% CO_2_, 5% O_2_, and 90% N_2_ at 38.5°C. Then, the embryos were transferred into 50 μl of mPZM-3 for further culture. In the second series of experiments, the embryos were cultured in the presence of 500 nM scriptaid, a histone deacetylase inhibitor, which enhanced the cloning efficiency of miniature pig [24], instead of cytochalasin B.

### Transfer of embryos

Female Clawn miniature pigs, aged 6–12 months, were used as recipients. Animals were pre-medicated with a cocktail of ketamin, medetomidine, and butorphanol tartrate mixture. Once sedated, animals were intubated and maintained with 1–4% isoflurane continuous inhalation. Embryo transfer was performed under general anesthesia through a midline incision 31 h after hCG injection (500 IU ASKA Pharmaceutical, Tokyo, Japan), as described previously [25]. Morphologically normal embryos (1- or 2-cell stage) were selected and surgically transferred into both sides of the recipients’ oviducts. At the same time, the non-transgenic SCNT embryos derived from kidney epithelial cells of another miniature boar were transferred as support of pregnancy. To test for pregnancy, abdominal ultrasonography was performed 28 d after embryo transfer, and approximately every 20 d thereafter. The surrogates were examined daily to detect estrus, and pregnancies were allowed to go to term.

### Microsatellite marker analysis

Genomic DNA was extracted from piglets’ umbilical cord, donor cells, and from uterine tissue using the DNeasy Total DNA kit (Qiagen, Chatsworth, CA, USA). We genotyped 12 microsatellite markers on the USDA swine linkage maps: S0038, S0091, S0227, SW1378, SW1434, SW1681, SW1954, SW2108, SW2494, SWR153, SWR1941, and TNFB [26]. PCR was performed using AmpliTaq Gold DNA polymerase (Applied Biosystems, Tokyo, Japan). Amplified DNA fragments were electrophoresed using an ABI 3100 genetic analyzer. The genotypes were analyzed with software from Applied Biosystems.

### RT-PCR analysis of human apo(a) expression

Portions of tissues isolated from three cloned piglets were collected. All samples were immediately frozen in liquid nitrogen and stored at −80°C until RNA isolation. Total RNA was isolated from tissue samples using the Fast Pure RNA Kit (Takara Bio, Otsu, Japan). The RT reaction and PCR reaction were carried out using a GeneAmp RNA PCR Kit (Applied Biosystems). The primers for apo(a) were 5’-TCCCTCTCTGTGCATCCTCT-3’ (forward) and 5’-GCCTCCACAGAAGTGCTTTC-3’ (reverse) [4]. Pig β-actin was used as an internal control for the analysis. The primers were 5’-GGACTTCGAGCAGGAGATGG-3’ (forward) and 5’-GCACCGTGTTGGCGTAGAGG-3’ (reverse) [27]. RT-PCR products were analyzed by electrophoresis on a 2.5% agarose gel (NuSieve GTG Agarose; Lonza, Walkersville, MD, USA). An apo(a)-positive stable transfectant clone was used as a positive control. Distilled water was also subjected to RT-PCR as a negative control.

### Immunohistochemical analysis of transgene expression in tissues from cloned piglets

Tissues were collected from each cloned piglet and fixed with 10% (w/v) formalin-PBS. After fixation, tissues were processed for immunoperoxidase staining as described in [28]. Peroxidase activity was visualized with DAB-H_*2*_O_*2*_. The sections were stained in hematoxylin, briefly washed in water, and gradually dehydrated through an ethanol gradient followed by xylenes. Mounted samples were examined using a Nikon (ECLIPSE 80i) microscope (Nikon, Tokyo, Japan).

### Analysis of plasma lipids and lipoproteins

Analyses of Lp(a), total cholesterol, triglycerides, LDL cholesterol, and HDL cholesterol were performed by SRL (Tokyo, Japan).

### Statistics

Data are presented as means ± standard error of the mean (SEM). All percentage data were arcsine-transformed. Transformed values and number of cells in the blastocysts were assessed by one-way analysis of variance followed by Fisher's protected least significant difference (LSD) as a post hoc test; *p* values < 0.05 were considered to be statistically significant.

## Results

### Vector construction

In a line of mice expressing the human apo(a) transgene under the control of the murine transferrin promoter, apo(a) circulating in the plasma associates with human LDL injected intravenously [7]. Therefore, complex formation of apo(a) with LDL must take place in the plasma following secretion, and it is not necessary for apo(a) to be produced in the liver. We designed the experiments using the nuclei of kidney epithelial cells as donor nuclei in SCNT. Consequently, we used the CAG promoter, a strong promoter that allows ubiquitous expression of the transgenes [29], instead of the transferrin promoter, a liver-specific promoter, used previously for production of apo(a) transgenic mice [7] and rabbits [8]. The vector used in the present study contains the CAG promoter, the HA-tagged apo(a) gene, the β-globin polyadenylation signal (polyA), and the neo gene under the control of herpes simplex virus thymidine kinase promoter (Fig 1A). The CAG promoter allows strong expression of the apo(a) protein, and the neo gene provides resistance to G418.

### Isolation of pig kidney epithelial cells stably expressing human apo(a)

Using pulse-chase experiments, Wang et al. established that more than 80% of apo(a) synthesized by hepatocytes was degraded prior to secretion, independently of coexpression of human apoB [30]. Thus, low secretion efficiency appears to be a general characteristic of human apo(a) proteins. Newly synthesized apoB also remains stably associated with the endoplasmic reticulum (ER) membrane; consequently, immunofluorescence staining reveals co-localization with ER markers [31]. Therefore, immunostaining of cells could be used for identification of cells expressing apo(a).

Prior to attempting to establish pig cells stably expressing human apo(a), we introduced the expression vector into MDCK cells. MDCK cells are epithelial cells derived from dog kidney, and have been used for isolation of stable transfectants expressing various different proteins [32]. We could isolate stable MDCK transfectants expressing human apo(a). When the cells were fixed with formaldehyde, permeabilized with Triton X-100, and then stained with anti-HA antibody, the intracellular perinuclear regions corresponding to the ER of MDCK cells were stained (Fig 1B). When MDCK cells expressing HA-tagged mouse E-cadherin (a cell–cell adhesion molecule) were stained with anti-HA antibody, they exhibited distinct membrane staining. Thus, immunostaining of cells can be used for identification of cells expressing apo(a). Furthermore, human apo(a) expressed under the control of the CAG promoter was not toxic in MDCK cells. We then introduced the same expression vector into pig kidney epithelial cells, isolated independent clones after selection with G418, and then immunostained with anti-HA antibody to identify apo(a)-positive clones. One of the clones isolated was analyzed further (Fig 1C). Immunoblot analysis of cell lysates with anti-HA antibody revealed that cells positive for immunostaining express a high–molecular weight protein of more than 400 kDa, whereas parental pig kidney epithelial (PKE) cells do not (Fig 1D). When the culture media were collected and subjected to immunoprecipitation with anti-HA antibody and immunoblot with anti-apo(a) antibody, the high–molecular weight protein was detected in the medium of apo(a) positive cells, but not in the medium of parental PKE cells (Fig 1E).

### In vitro developmental ability and transgene expression of SCNT embryos

**Fig 2:**
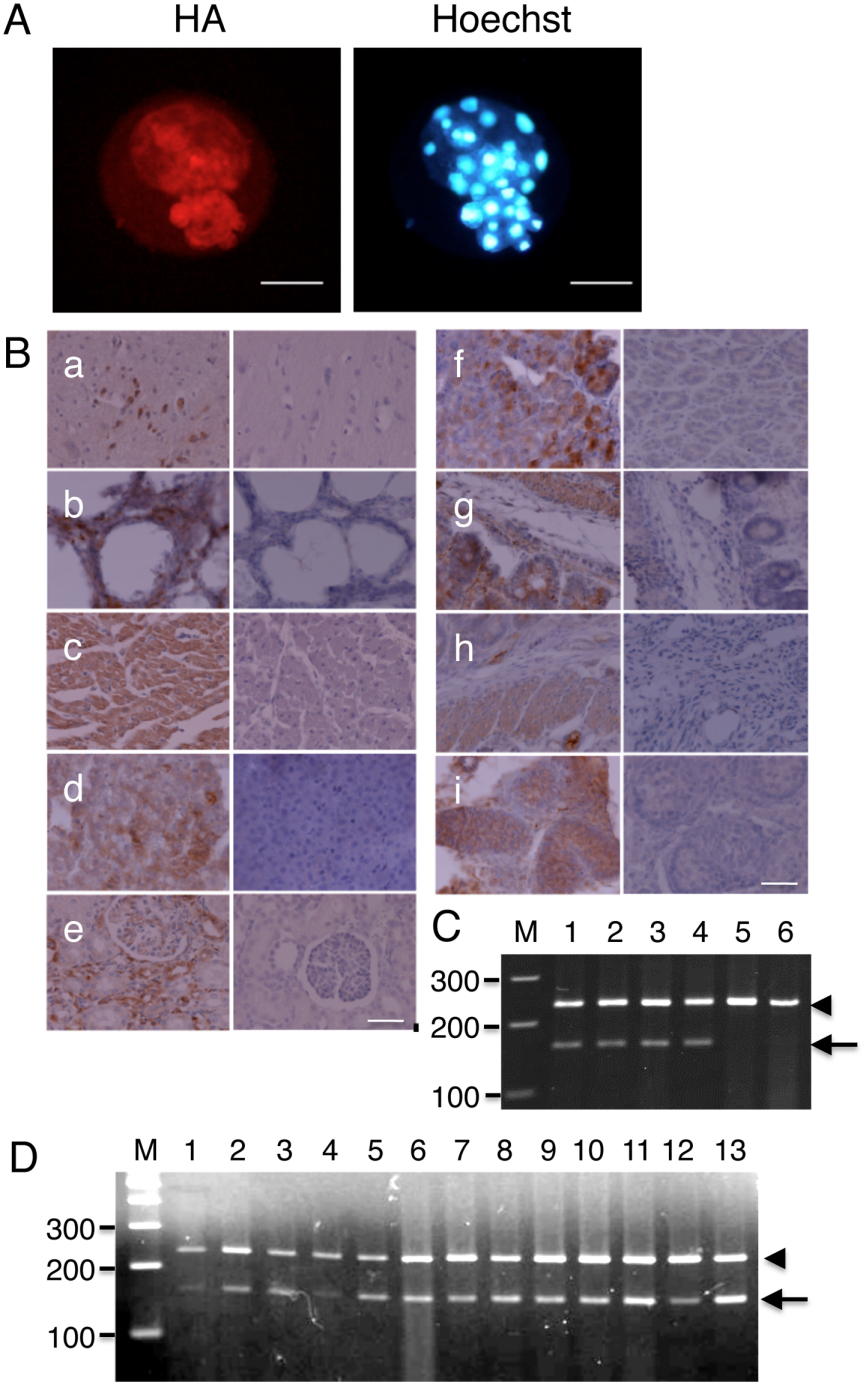
Expression of human apo(a) in SCNT embryos and tissues of transgenic piglets. A, SCNT embryos after 7 d culture were stained with anti-HA antibody (left) or Hoechst 33342 (right). Bars, 50 μm. B, Paraffin sections were stained with anti-HA antibody, followed by HRP-conjugated secondary antibody and DAB substrate. Finally, the sections were stained with hematoxylin and mounted. The left and right panels show tissue from transgenic and non-transgenic piglets, respectively. a) brain, b) lung, c) heart, d) liver, e) kidney, f) stomach, g) small intestine, h) large intestine, i) testis. Bars, 100 μm. C, RT-PCR analysis showing expression of human apo(a) mRNA in the kidney of three transgenic piglets. Lanes: M, 100 bp DNA ladder markers; 1, kidney of transgenic piglet #1; 2, kidney of transgenic piglet #2; 3, kidney of transgenic piglet #3; 4, pig kidney epithelial cells stably expressing human apo(a) used as donor of SCNT; 5, kidney of non-transgenic pig; 6, liver of non-transgenic piglet. A band of 233 bp, corresponding to the β-actin (arrowhead) was detected in all samples, whereas a band of 158 bp, corresponding to the human apo(a) transcripts (arrow), is found in transgenic samples but not in non-transgenic samples. D, RT-PCR analysis showing expression of human apo(a) mRNA in the organs of a transgenic piglet. Bands of 233 bp (β-actin, arrowhead) and 158 bp (human apo(a), arrow) are visible. Lanes: M, 100 bp DNA ladder markers; 1, brain; 2, lung; 3, heart; 4, aorta; 5, muscle; 6, liver; 7, kidney; 8, pancreas; 9, spleen; 10, stomach; 11, small intestine; 12, large intestine; 13, skin.

Experiments were repeated four times. The fusion rate of SCNT embryos reconstituted with transgenic kidney epithelial cells was 77.8% (n = 200). The rate of cleavage, blastocyst formation rate, and mean blastocyst cell number were 80.2 ± 4.3%, 18.8 ± 3.9%, and 39.7 ± 9.4, respectively. Furthermore, all blastocysts (n = 38) expressed human apo(a) as assessed by immunohistochemistry (Fig 2A). Thus, the selected donor cells do not undergo the epigenetic reprogramming that would take place under early embryonic conditions.

### Post-transfer developmental ability of transgenic SCNT embryos

In the first series of experiments, cloned embryos were transferred into the oviducts of five recipient gilts (Table 1). Two recipients became pregnant as determined by ultrasound at 28 d post-transfer. One recipient maintained pregnancy to term, and three male piglets were delivered via Cesarean section 111 d after embryo transfer. The piglets weighed 220, 220, and 200 g and exhibited normal morphology. Genetic analysis confirmed that the piglets were identical to the cell line used for nuclear transfer at all 12 polymorphic microsatellite loci examined (S1 Table). The other pregnant recipient gilt aborted at mid-gestation, and no piglets were obtained from that pregnancy. All cloned piglets died of unknown causes 1–7 d after birth, but were used in the following experiments.

**Table 1:** *In vivo* development ability of SCNT embryos derived from transgenic kidney epithelial cells.

Recipient	Number of embryos transferred^a^	Pregnancy diagnosis by ultrasonography^b^	Number of piglets born	Birth weight of piglet (g)^c^ ^,^ ^d^
A	106 (108)	Yes	0	-
B	60 (146)	No	-	-
C	210 (0)	Yes	3	220, 220, 200
D	125 (124)	No	-	-
E	105 (102)	No	-	-

^a:^ Numbers in parentheses indicate the number of non-transgenic SCNT embryos transferred to support the pregnancy.

^b:^ Detection of fetal sac and/or fetus.

^c:^ The birth weight of the three piglets was considerably less than the average birth weight (500 g) of the Clawn miniature pig. Therefore, they might be premature and we could not save their life.

^d:^ We do not know the reason why four co-transfers of transgenic SCNT embryos and non-transgenic ones produced no viable transgenic piglets whereas the one transfer of transgenic embryos by themselves produced viable piglets. One possible explanation may be as follows. We use around 200 embryos for one transfer, and when the number of transgenic embryos prepared during a certain period of time does not reach 200, we includeed non-transgenic ones. In the case when we could prepare more than 200 transgenic embryos, we did not include non-transgenic ones. In the latter case, their quality was good enough for pregnancy.

### Analysis of transgene expression by immunohistochemical observation and RT-PCR of tissues from cloned piglets

As shown in Fig 2B, human apo(a) was detected in all tissues of cloned piglets, but not in a control piglet. This observation was confirmed by RT-PCR analysis (Fig 2C). This broad pattern of expression was expected, because the CAG promoter used to drive apo(a) expression is active in a variety of cells.

### Establishment of a transgenic line that exhibited a high level of apo(a) in the plasma and stable transmission of the transgene to the offspring

**Fig 3:**
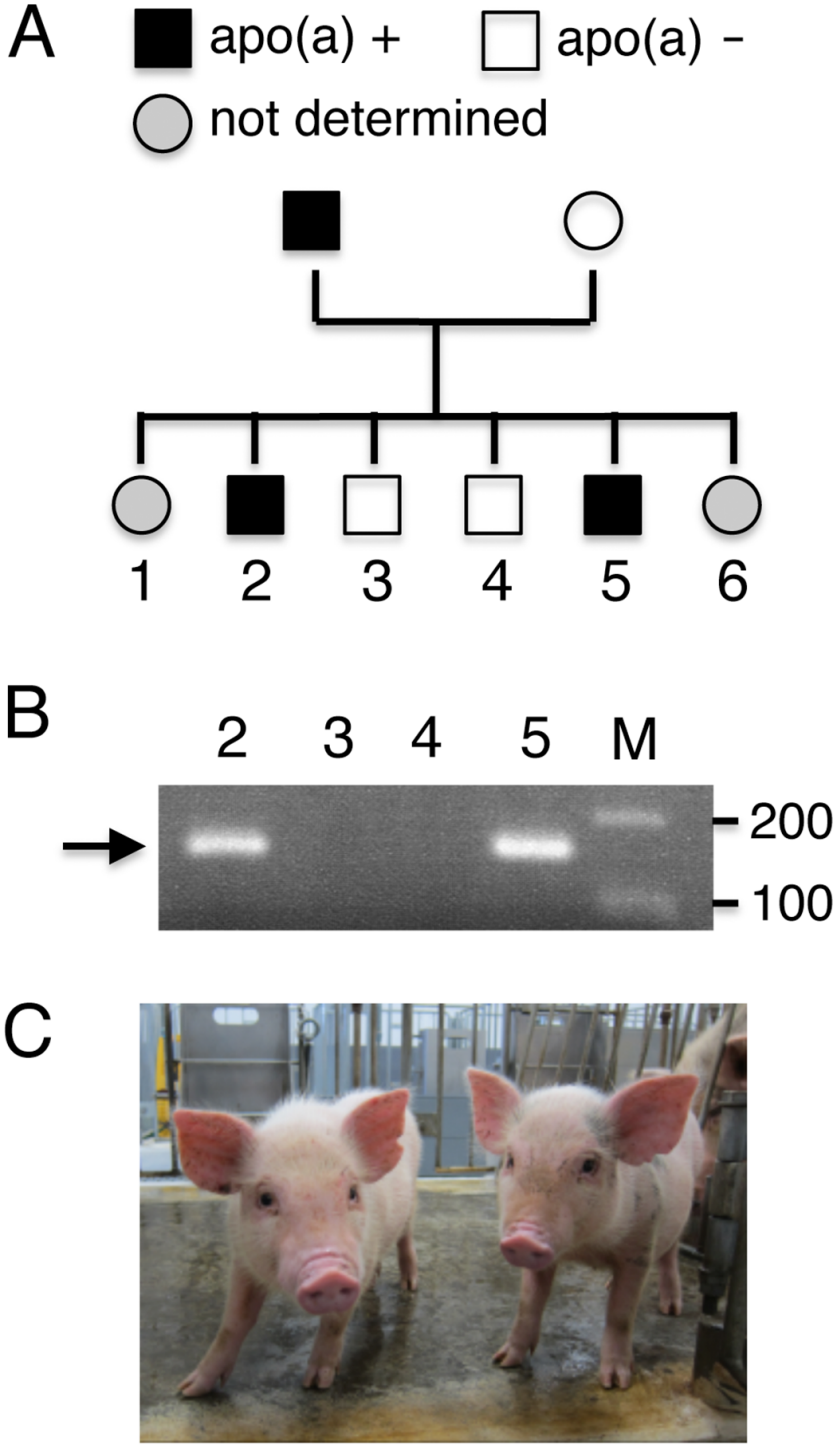
The transgenic miniature pig and his offspring. A, pedigree of the transgenic miniature pig clone expressing human apo(a). Two offspring (No. 1 and 6) were dead when they were found, and could not be analyzed. B, Identification of the transgene, apo(a), in genomic DNA of offspring by PCR. The arrow indicates the position of the PCR product. C, Photograph of apo(a)-positive offspring (No. 2 and 5).

In the second series of experiments, we finally succeeded in obtaining a piglet that is PCR-positive for human apo(a) and exhibited a high level of Lp(a) in plasma (Table 2). This piglet was used as a founder to establish a apo(a)-transgenic line of miniature pigs (Fig 3A). Six piglets were produced from a sow after artificial insemination using semen from the PCR-positive boar (Minidora), of which two died for unknown reasons, but seemed to be killed by their mother by crashing, and could not be analyzed. Two of the four living piglets were positive in PCR analysis, whereas the other two were negative (Fig 3A, 3B, and 3C). The levels of Lp(a) in the PCR-positive founder and two PCR-positive offspring on a standard chow diet were 682 mg/dL, 427 mg/dL, and 514 mg/dL, respectively (Table 2). These values are much higher than the levels of Lp(a) in three nontransgenic female pigs and the two PCR-negative offspring. Expression of human apo(a) in transgenic pigs did not result in a significant change in plasma total cholesterol, triglycerides, LDL-C levels, or HDL-C levels (Table 2).

**Table 2:** Plasma lipid and human apo(a) levels in transgenic miniature pigs on chow diet.

Miniature pig	158-bp apo(a) PCR band	Lp(a) mg/dL	Total Cholesterol mg/dL	TG mg/dL	LDL-C mg/dL	HDL-C mg/dL
Minidora (♂)	+	682	77	16	37	38
Natts (♀)	nd	2	64	17	33	30
CH53 (♀)	nd	2	91	33	45	40
Kinoko (♀)	nd	<1	60	33	27	32
No. 2 (♂)	+	427	57	16	33	22
No. 3 (♂)	-	<1	67	7	31	34
No. 4 (♂)	-	<1	51	49	22	28
No. 5 (♂)	+	514	78	15	38	39

Note: Minidora is the founder of the human apo(a)-transgenic miniature pig line. Three non-transgenic females (Natts, CH53, and Kinoko) were used as negative controls. Four piglets (No. 2, No. 3, No. 4, and No. 5) were produced from a sow after artificial insemination using semen from the PCR-positive boar (Minidora), of which two were positive in the PCR analysis (No. 2 and No. 5). Lp(a), lipoprotein(a); LDL-C, LDL cholesterol, HDL-C, HDL cholesterol; TG, triglycerides. nd, not determined.

**Fig 4:**
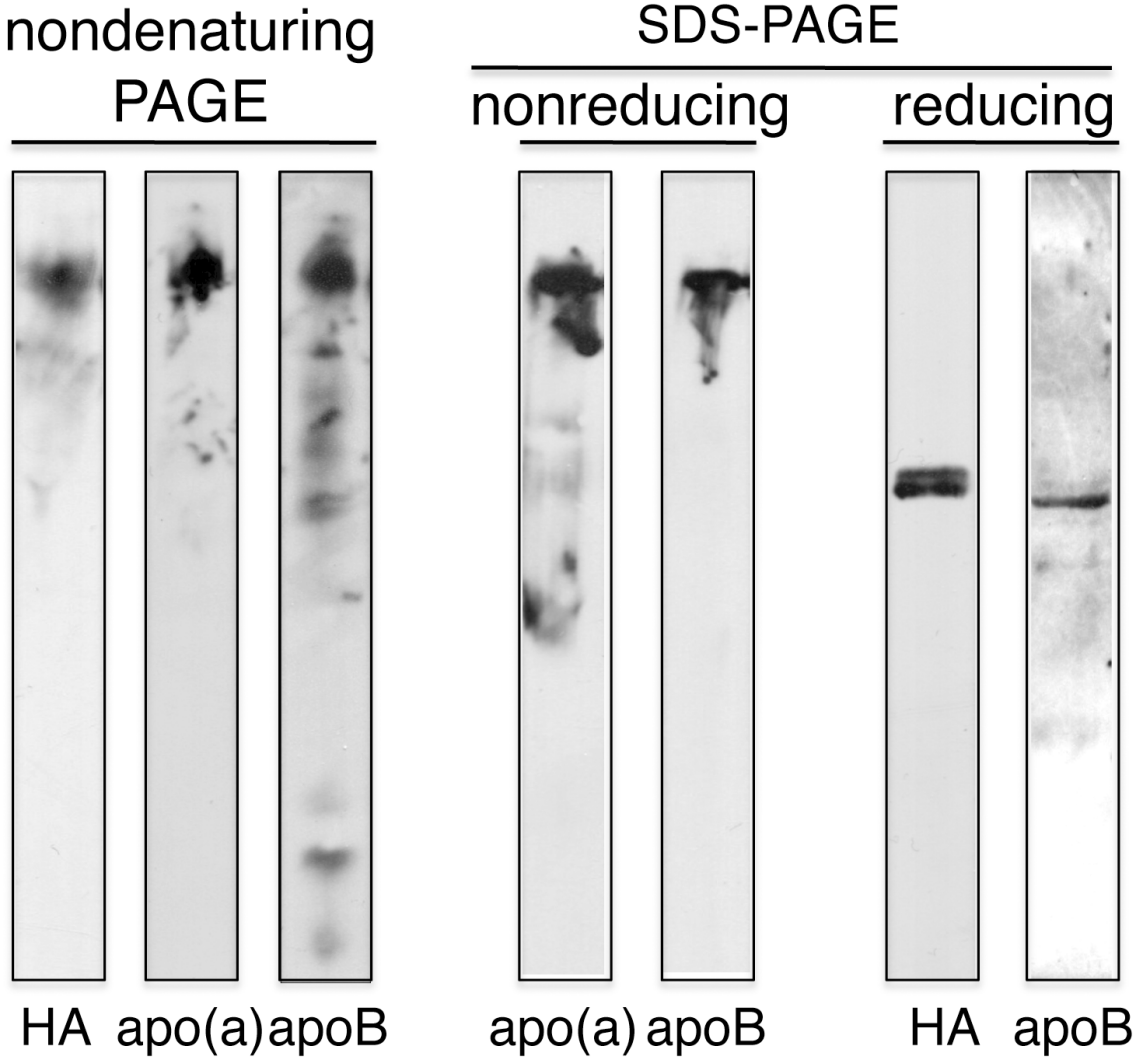
Immunoblot analysis of transgenic pig plasma apo(a). Aliquots of plasma were separated by either 4% nondenaturing polyacrylamide gel electrophoresis (PAGE) or 4% SDS-PAGE under nonreducing (left) or reducing (right) conditions. The SDS-PAGE samples of nonreducing and reducing conditions were electrophoresed on the same gels. After electrophoretic transfer, the proteins were immunoblotted using an anti-human apo(a) mAb or an anti-apoB mAb as described in Materials and Methods. Since the reactivity of materials separated under reducings to anti-apo(a) mAb become weak, they were blotted with anti-HA mAb.

In the human Lp(a) particle, apo(a) is covalently linked to apoB-100 by the presence of a single disulfide bridge [33]. It has been demonstrated that apo(a) and apoB-100 are present in a 1:1 molar ratio in Lp(a) [34]. To determine whether the apo(a) in the pig plasma was incorporated into a lipoprotein particle, total lipoproteins were subjected to electrophoresis on a 4% nondenaturing polyacrylamide gel (Fig 4, left panels). The apo(a) immunoreactive material in pig plasma migrated to a position that was identical to the position of apoB recognized by anti-apoB antibody. The HA tag was added at the *C*-terminus of apo(a) and the HA immunoreactive material in pig plasma also migrated to the position of apoB. Co-migration of apo(a) and apoB strongly suggested that they were in the same complex. To determine whether apo(a) was disulfide linked to pig apoB, the pig plasma were subjected to electrophoresis on a 4% denaturing polyacrylamide gel (SDS-PAGE) after boiling in the presence 2.3% SDS alone (nonreducing conditions) or in the presence of 2.3% SDS and 5% 2-mercaptoethanol (reducing conditions) (Fig 4, right panels). When the plasma samples were analyzed under nonreducing conditions, apo(a) migrated to a position that was identical to the position of apoB. Since after reduction with 2-mercaptoethanol, the reactivity of the materials to anti-apo(a) antibody was significantly reduced, we used anti-HA antibody to detect apo(a) under reducing condition. The epitope may be dependent on disulfide bonds. Under reducing conditions, two closely migrating immunoreactive bands and a single slightly faster migrating band were detected with anti-HA and anti-apoB antibodies, respectively. These bands migrated much faster than the bands detected under nonreducing conditions. These results suggest that the apo(a) in the transgenic pig circulates as a complex disulfide linked to apoB in plasma.

## Discussion

Apo(a)-transgenic mice and rabbits have been successfully used in studies addressing the role of Lp(a) in atherosclerosis and thrombosis. However, large-animal models that recapitulate human disease pathophysiology are needed for translational research in atherosclerosis, and genetic engineering of well-characterized miniature pig strains has the potential to generate such models. In this study, we created a porcine model that expresses human apo(a) in Clawn miniature pigs, which showed high levels of human Lp(a) in plasma.

Previous studies have identified elevated plasma Lp(a) concentrations (> 30 mg/dL) as a risk factor for a variety of atherosclerotic and thrombotic vascular diseases including peripheral vascular disease, venous thromboembolism, recurrent thrombotic stroke in children, and coronary heart disease (CHD) [35]. Lp(a) levels in human apo(a) transgenic rabbits are ~4.5 mg/dL [8], equivalent to relatively low levels in humans. Despite the presence of Lp(a) particles in the rabbits, which do not normally express apo(a), transgenic rabbits that were fed a chow diet for up to 2 years did not develop atherosclerotic lesions [9]. This is consistent with the notion that a low level of Lp(a) per se is not atherogenic. On a cholesterol-rich diet, however, the transgenic rabbits developed more extensive atherosclerotic lesions in the aorta and other arteries than did control rabbits [9], in agreement with studies of human apo(a) transgenic mice [7]. On the other hand, Berg et al. [36], studying relatively aged mice (average age, 66 weeks at sacrifice) that had been fed normal chow throughout their lifetime, found that transgenic apo(a) mice had a greater extent of (spontaneous) atherosclerotic lesion formation than nontransgenic control mice. Thus, transgenic apo(a) mice on a normal diet could be a useful animal model for the study of spontaneous human atherosclerosis, its treatment, and prevention. The human apo(a) transgenic miniature pigs produced in the present study expressed > 400 mg/dL of human Lp(a) in plasma, much higher than the plasma concentrations of Lp(a) in apo(a) transgenic mice and rabbits (8.5 mg/dL and ~4.5 mg/dL, respectively) [7, 8]. Therefore, it is of great interest to determine whether the apo(a) transgenic miniature pig develops spontaneous atherosclerosis, and an atherogenic diet is required for development of atherosclerosis.

In humans, a blood Lp(a) level higher than 50 mg/dL is regarded as a very high risk. In the current model, Lp(a) levels are higher than 400 mg/dL. At present we can not discuss the relevance of the model's high Lp(a) level to human's scenario. Further studies are required.

Transgenic pigs have been produced by SCNT techniques using donor cells derived from fetuses and young pig fibroblasts [37,38]. In the present study, we used epithelial cells isolated from a boar (positive for epithelial markers E-cadherin and cytokeratin 5 and 8, S1 Fig) as donor cells. We introduced the human apo(a) gene into kidney epithelial cells, and used their nuclei as donor nuclei in SCNT, ultimately generating transgenic miniature pigs that expressed human apo(a). Furthermore, the fusion rate of SCNT embryos reconstituted from transgenic kidney epithelial cells and the *in vitro* developmental capacity of SCNT embryos were comparable to those other genetically modified somatic cells used as donors in our laboratory [22]. This observation suggests the possibility of using kidney epithelial cells as donor cells for genetic modification.

The advantage of using SCNT to make genetically modified cloned animals is that precise genetic modifications can be accomplished and confirmed in cultured cells prior to generating the animal. This is very important, because true embryonic stem cells have not yet been isolated from any domestic animal [39]. Expansion of NT techniques for domestic animals is rapidly progressing through the utilization of SCNT to create genetically modified cloned animals. The resultant clones tend to exhibit inefficient transmission of the transgene and a significant level of phenotypic instability. This inefficient transmission of the transgene may be due to the restricted lifespan of somatic donor cells in culture, which can limit the time available for the selection of donor cells. The instability may be due to epigenetic reprogramming and/or genomic damage in the donor cells [40]. We overcame these problems in the following ways. First, we used immunofluorescence staining of the transfected epithelial cells for the selection of apo(a)+ donor cells used to generate the cloned pigs. The method allowed us to identify transgenic epithelial clones stably and homogenously expressing apo(a) during culture. The use of this screening method markedly reduced the time spent by the transgenic cells in culture, a factor that influences chromosomal condition and the success of SCNT [41]. The low passage number of the apo(a)+ transgenic cells used for SCNT may have contributed to the successful production of a viable cloned litter. The same analysis of SCNT blastocysts revealed that all blastocysts expressed human apo(a). Thus, the selected donor cells did not undergo the epigenetic reprogramming that would ordinarily take place under early embryonic conditions. The three piglets obtained from a sow in a first series of experiments died of unknown causes 1–7 d after birth, they might be premature, because their birth weight was considerably less than the average birth weight of the Clawn miniature pig. Ultimately, we succeeded in establishing a transgenic clone in the next series of experiments. Use of scriptaid, a histone deacetylase inhibitor, which improve the cloning efficiency of SCNT embryos of miniature pig [24], in the second series of experiments may have improved the outcome.

Recently, Wei et al. [42] reported the generation of a human apolipoprotein CIII [apo(CIII)]-transgenic miniature pig model. Furthermore, Al-Mashhadi et al. [43] reported the creation of Yucatan minipigs with liver-specific expression of human D374Y-PCSK9. D374Y-PCSK9–transgenic pigs exhibited reduced hepatic LDL receptor levels, impaired LDL clearance, severe hypercholesterolemia, and spontaneous development of progressive atherosclerotic lesions that could be visualized by noninvasive imaging. In this study, we successfully generated transgenic miniature pigs expressing human apo(a). Because of the similarities between pig and human cardiovascular anatomy and physiology, second-generation transgenic animal models using miniature pigs will become very valuable. Human apo(a) transgenic miniature pigs represent a potentially useful model for evaluating the efficacy and pharmacology of new drugs for atherosclerosis.

## Supporting Information

S1 Fig PKE cells show epithelial cell nature. A, Immunoblot detection of E-cadherin in MDCK and PKE cells. Cells were directly lysed in SDS-sample buffer, subjected to SDS-PAGE, and examined by immunoblotting with E-cadherin antibodies. B, Immunofluorescence staining of MDCK and PKE cells with cytokeratin antibodies. Cells were cultured on coverslips for 24 h and then examined by immunofluorescence microscopy for cytokeratin 5/8. Bars, 25 μm.
(TIF)

S1 Table Analysis of microsatellite markers in denomic DNA from piglets, nuclear donor cells. (DOCX)
