# Supplementary figures and images for: Production of Cloned Miniature Pigs Expressing High Levels of Human Apolipoprotein(a) in Plasma
Source: PLoS One. 2015 Jul 6;10(7):e0132155. doi: 10.1371/journal.pone.0132155 (PMC4492603; doi:10.1371/journal.pone.0132155)

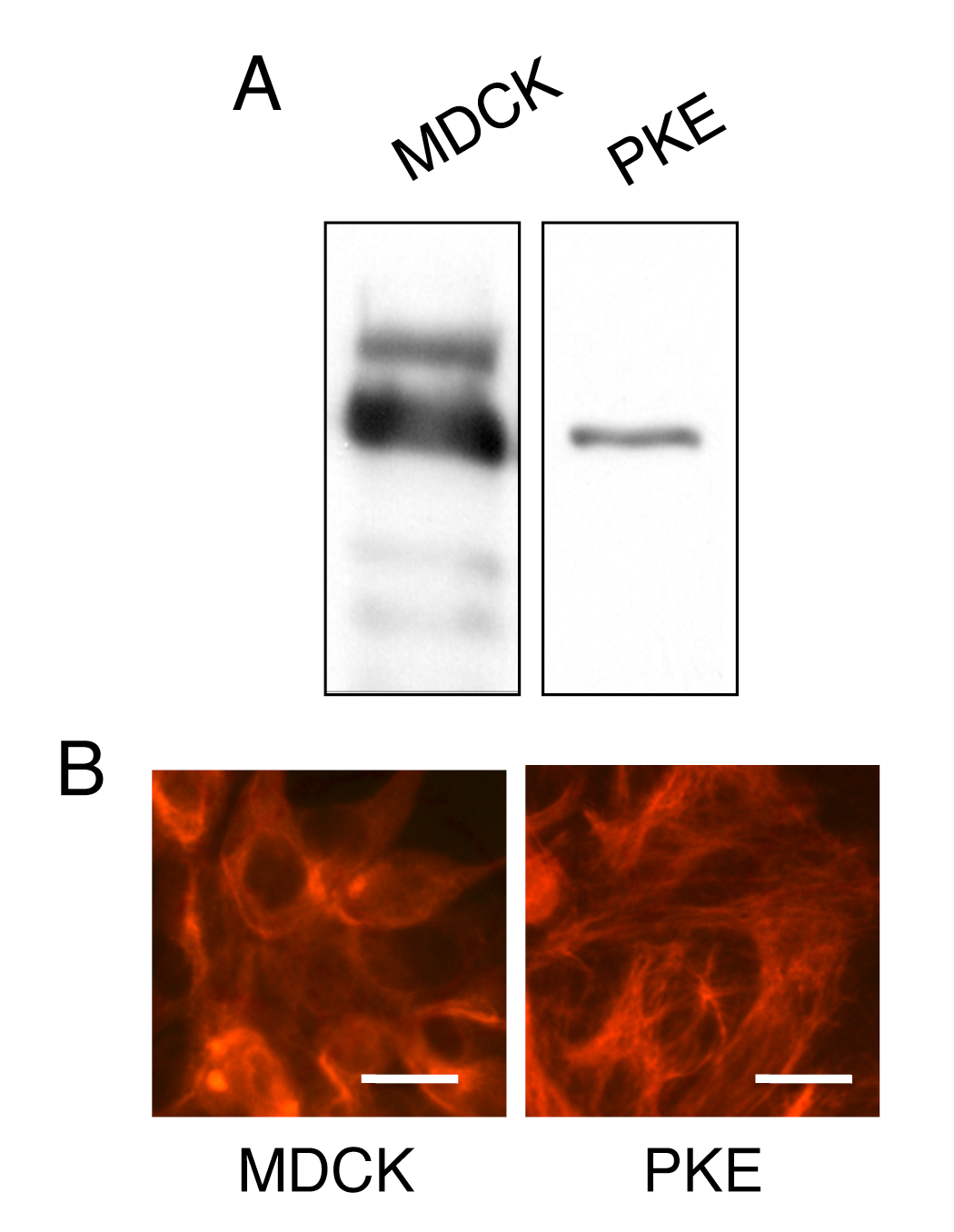

Supplement: S1 Fig — A, Immunoblot detection of E-cadherin in MDCK and PKE cells. Cells were directly lysed in SDS-sample buffer, subjected to SDS-PAGE, and examined by immunoblotting with E-cadherin antibodies. B, Immunofluorescence staining of MDCK and PKE cells with cytokeratin antibodies. Cells were cultured on coverslips for 24 h and then examined by immunofluorescence microscopy for cytokeratin 5/8. Bars, 25 μm. (TIF) [file pone.0132155.s001.tif]
