# Supplementary material for: Production of Cloned Miniature Pigs Expressing High Levels of Human Apolipoprotein(a) in Plasma
Source: PLoS One. 2015 Jul 6;10(7):e0132155. doi: 10.1371/journal.pone.0132155 (PMC4492603; doi:10.1371/journal.pone.0132155)
Supplement: S1 Table — (DOCX) [file pone.0132155.s002.docx]

| **Table S1. Analysis of microsatellite markers in denomic DNA from piglets, nuclear donor cells** | | | | | |
| --- | --- | --- | --- | --- | --- |
|  | Allele sizes | | | | |
| Marker | Alive piglet　No.1 | Alive piglet　No.2 | Alive piglet　No.3 | Donor cells | |
| S0038 | 135/141 | 135/141 | 135/141 | 135/141 | |
| S0091 | 148/160 | 148/160 | 148/160 | 148/160 | |
| S0227 | 238/238 | 238/238 | 238/238 | 238/238 | |
| SW1378 | 88/88 | 88/88 | 88/88 | 88/88 | |
| SW1434 | 146/146 | 146/146 | 146/146 | 146/146 | |
| SW1681 | 122/135 | 122/135 | 122/135 | 122/135 | |
| SW1954 | 165/195 | 165/195 | 165/195 | 165/195 | |
| SW2108 | 140/140 | 140/140 | 140/140 | 140/140 | |
| SW2494 | 96/96 | 96/96 | 96/96 | 96/96 | |
| SWR153 | 227/227 | 227/227 | 227/227 | 227/227 | |
| SWR1941 | 217/217 | 217/217 | 217/217 | 217/217 | |
| TNFB | 178/178 | 178/178 | 178/178 | 178/178 | |
| Analysis confirmed that the piglets were identical to the cell line used for nuclear transfer at all 12 polymorphic microsatellite loci examined. | | | | |  |
